# Using participatory action research to improve immunization utilization in areas with pockets of unimmunized children in Nigeria

**DOI:** 10.1186/s12961-021-00719-9

**Published:** 2021-08-11

**Authors:** Ngozi N. Akwataghibe, Elijah A. Ogunsola, Oluwafemi A. Popoola, Adanna I. Agbo, Marjolein A. Dieleman

**Affiliations:** 1 Royal Tropical Institute, Amsterdam, The Netherlands; 2 Athena Institute, Vrije Universiteit Amsterdam, Amsterdam, The Netherlands; 3 Ogun State Primary Health Care Development Board, Abeokuta, Nigeria; 4 College of Medicine, University of Ibadan, Ibadan, Nigeria; 5 Morgan State University, Baltimore, MD USA

**Keywords:** Participatory action research, Routine immunization, Community, Health workers, Government

## Abstract

**Background:**

In 2005, Nigeria adopted the Reaching Every Ward strategy to improve vaccination coverage for children 0–23 months of age. By 2015, Ogun state had full coverage (100%) in 12 of its 20 local government areas, but eight had pockets of unimmunized children, with the highest burden (37%) in Remo North. A participatory action research (PAR) approach was used to facilitate implementation of local solutions to contextual barriers to immunization in Remo North. This article assesses and seeks to explain the outcomes of the PAR implemented in Remo North to understand whether and possibly how it improved immunization utilization.

**Methods:**

The PAR intervention took place from 2016 to 2017. It involved two (4-month) cycles of dialogue and action between community members, frontline health workers and local government officials in two wards of Remo North, facilitated by the research team. The PAR was assessed using a pre/post-intervention-only design with mixed methods. These included household surveys of caregivers of 215 and 213 children, respectively, 25 semi-structured interviews with stakeholders involved in immunization service delivery and 16 focus group discussions with community members. Data were analysed using the Strategic Advisory Group of Experts (SAGE) vaccine hesitancy framework.

**Results:**

Collaboration among the three stakeholder groups enabled the development and implementation of solutions to identified problems related to access to and use of immunization services. At endline, assessment by card for children older than 9 months revealed a significant increase in those fully immunized, from 60.7% at baseline to 90.9% (*p* < .05). A significantly greater number of caregivers visited fixed government health facilities for routine immunization at endline (83.2%) than at baseline (54.2%) (*p* < .05). The reasons reported by caregivers for improved utilization of routine immunization services were increased community mobilization activities and improved responsiveness of the health workers. Spillover effects into maternal health services enhanced the use of immunization services by caregivers. Spontaneous scale-up of actions occurred across Remo North due to the involvement of local government officials.

**Conclusion:**

The PAR approach achieved contextual solutions to problems identified by communities. Collection and integration of evidence into discussions/dialogues with stakeholders can lead to change. Leveraging existing structures and resources enhanced effectiveness.

**Supplementary Information:**

The online version contains supplementary material available at 10.1186/s12961-021-00719-9.

## Background

Immunization is one of the most effective and efficient health interventions, critical to the reduction of morbidity and mortality among children under 5 years old [1]. Though the global immunization trends over the years have been positive, diphtheria, tetanus and pertussis (DTP)3 coverage remained the same from 2015 to 2019 (85%), with about 19.7 million children still vulnerable to vaccine-preventable diseases [2]. Most of those children are found in 10 countries, including Nigeria [1].

In 2005, Nigeria adopted the Reaching Every Ward (REW) strategy to improve vaccination coverage for children 0–23 months of age. The 2018 Nigeria Demographic Health Survey showed that only 31% of children aged 0–23 months had completed a full course of prescribed routine immunization—with differences across geopolitical zones ranging from 76% in the South East to 5% in the North West [3]. In 2015, Ogun state in Nigeria had recorded full coverage across 12 of its 20 local government areas (LGAs). However, eight LGAs had pockets of unimmunized children, with the highest burden (37%) in the Remo North LGA. Since the exact factors responsible for this trend were not known, a participatory action research (PAR) strategy was implemented to address the problem of poor immunization coverage in parts of Ogun state. The rationale was that using methods that involve iterative processes of reflection and action carried out jointly with communities, health workers and local government officials would likely provide insight into the relevant problems and their realistic, context-specific solutions.

PAR emphasizes collective enquiry and research, based on experience and societal history [4] and broadly consists of a cyclical process of fact-finding, action and evaluation [5]. Several studies have used PAR successfully in addressing health issues in African countries including Kenya, Zambia and South Africa [6–9], but not specifically for immunization. However, in other contexts, Beauregard et al. [10] used PAR to improve timeliness of vaccination in children, while Willis et al. [11] and Crowley [12] used PAR to increase immunization rates. Theoretical concepts that led to the emergence of this type of research are broadly based on the principle that complex, persistent or unstructured problems cannot be tackled effectively by a more traditional research approach, which does not adequately address the underlying social, political, economic, cultural and ethical aspects of the problem [13–17]. A common ideology in all PAR designs is that research and action must be done “with” people and not “on” or “for” people [18–21].

The PAR consisted of research-informed dialogue and action cycles and was led by a policy-maker on the State Primary Health Care Development Board (SPHCDB). It was implemented in Ipara and Ilara wards of Remo North as part of the National Programme on Immunization. This article assesses and seeks to explain the outcomes of the PAR implemented in Remo North LGA of Ogun state in order to understand whether and how it improved immunization utilization in the two focal wards.

## Case description

Two rounds of dialogue and action took place between community women and men in Ipara and Ilara, frontline health workers in both wards, and Remo North local government officials. This was facilitated by the research team consisting of a policy-maker and academicians. Community members included caregivers of children under 5 years old; Christians, Muslims and Traditionalists; indigenous and non-indigenous groups; and representatives of the official REW social mobilization structures at the ward and local government levels—ward development committee (WDC) and social mobilization committee (SMC), respectively.

**Fig. 1:**
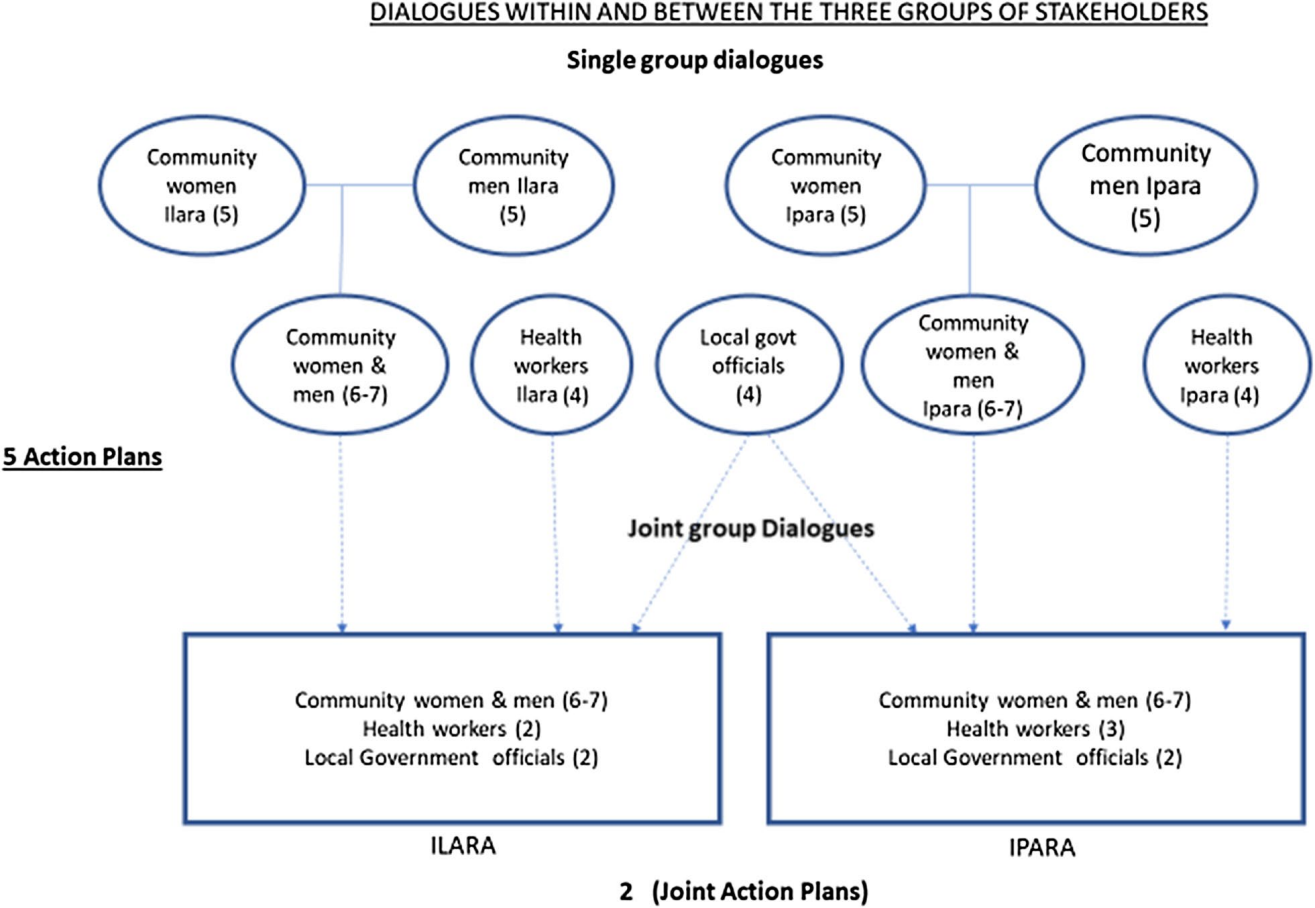
Single and joint groups dialogues

Results of a situational analysis at baseline (later published—https://www.frontiersin.org/articles/10.3389/fpubh.2019.00392/full) were presented to the three groups of stakeholders, validated by them and used in their discussions. The stakeholders identified problems influencing immunization coverage and their possible solutions, and developed joint action plans (JAPs) for change. The first round of dialogue took place in July 2016. Single-group dialogues were first held, and representatives from the single-group dialogues were nominated for further (joint group) dialogues. There were no financial incentives given to the three groups of stakeholders for participation in this study, but costs of transportation to and from the dialogue venue were reimbursed. Figure 1 illustrates the dialogue process.

In each ward, the actions and plans formulated per group were compared and discussed within the joint dialogue groups to develop JAPs. The joint dialogue participants thereafter referred to themselves as joint action committees (JAC). The JAPs were implemented by the JACs and WDCs in both wards. The first action phase lasted 4 months. Progress was tracked via the National Health Management Information System (NHMIS) and monitoring data, and the cycle repeated. The second round of dialogue took place in December 2016, and the second JAPs were implemented in another 4-month action phase. A summary of the JAC and the implementation of actions during the two action phases in the two wards is presented in Table 1.

**Table 1:** Summary of the JAC and implementation of actions

The JAC and implementation of actions during the two action phases	Ilara	Ipara
Community JAC members (chosen by the wider community group to represent them)	3 Women and 4 men Age range: 27–69 years Occupations* include clergy (1), native doctor (1), farmer (3), trader (1), hairdresser (1), traditional birth attendant (TBA) (1) WDC chairman chosen as JAC chairman	3 Women and 4 men Age range: 42–69 years Occupations* include clergy (2), trader (3), farmer (3), retired teacher (1) WDC chairman chosen as JAC chairman
Health workers in the JAC	Frontline health workers (2)	Frontline health workers (2)
Local government officials in the JAC	LGA officials in leadership positions and immunization service delivery (2)	LGA officials in leadership positions and immunization service delivery (2)
Implementation of the joint action plans—strategies and priorities	1. WDC in Ilara was re-established by community members and collaborated with the JAC to implement the JAPs 2. Community members carried out advocacy visits to the king—and renovated the health facility and cleared the environment 3. LGA officials achieved the deployment of two more health workers to the Ilara facility 4. Health workers (with WDC and religious leaders in the JAC) addressed knowledge and awareness via home visits and increased community mobilization activities 5. LGA officials and the health workers reinstituted antenatal care and delivery services in the Ilara facility	1. WDC collaborated with JAC to implement plans 2. The WDC and community members organized the purchase of a megaphone for community mobilization; conducted advocacy visits to nonindigenous groups and advocacy visits to the government to address health worker shortage 4. LGA officials and health workers carried out health promotion activities and ensured the availability of vaccines at scheduled times 5. LGA officials and health workers commenced delivery services for primigravid women (previously not available) in the health facility

## Methods

In this section we describe the study design, sampling and recruitment of the respondents, and the conceptual framework used in data analysis.

### Study design

We used a pre-test/post-intervention only approach to evaluate the outcomes of the PAR. Baseline (situational analysis) in May 2016 and endline assessments (in April 2017) were carried out using mixed methods comprising a household survey (HHS), secondary data analysis of the NHMIS, focus group discussions (FGD) and semi-structured interviews (SSI). We used concurrent mixed methods designs at baseline and endline—the quantitative and qualitative data were collected in parallel, within the same time frame. Integration was carried out during data analysis and interpretation of results. The qualitative interviews were used to explain the results of the survey and to gain more insight into contextual factors.

Quantitative methods included a survey at the household level targeting caregivers responsible for the vaccination of at least one under-five child, and secondary analysis of NHMIS data to track immunization coverage. Qualitative methods included FGDs with community men and women—used to explore the uptake of the intervention by the communities and changes in knowledge, attitudes and utilization of immunization. This was triangulated with SSIs of key stakeholders. The SSIs helped us explore system challenges and to understand whether there was a match (or mismatch) between community views and the views of other stakeholders. Baseline and endline data collection was carried out by a team of two quantitative and two qualitative researchers, 14 enumerators and eight qualitative research assistants.

### Sampling and recruitment

Remo North was purposively selected for this study because of the burden of unimmunized children. Two focal wards were selected. These were Ipara and Ilara, with high and low immunization coverage, respectively, according to the 2015 NHMIS data. We wanted to determine the range of facilitators and barriers to immunization, and whether there were differences among the sites which could explain the outcomes. In terms of characteristics, Ilara is essentially a remote and rural farm settlement, while Ipara is semirural, having more commercial activity and a more organized structure with numbered streets.

Enumeration of households for the survey was conducted by officials of the National Population Commission. This exercise identified houses with children under 5 years of age, who were the focus of enquiry. The HHS sampling was conducted using the World Health Organization (WHO) modified two-stage cluster sampling method [22]. Using probability proportional to size techniques, we identified the clusters for the study. Thirty clusters were selected across the two wards—12 in Ilara and 18 in Ipara. To identify households, in each cluster an arbitrary but central starting point was identified. Consecutive houses along this path were visited to identify households eligible for inclusion. One under-five child was selected from households in seven consecutive homes. Where more than one eligible child was present in a household, one was selected using a table of randomly generated numbers. All eligible children were selected in the seventh household of each cluster, as required by this method. The respondents in this study were caregivers of under-five children in the selected wards. Individuals were eligible if they were currently domiciled in the ward. Information was obtained primarily from the mother/primary caregiver. Interviews took between 25 and 40 minutes to complete. Most interviews were conducted in Yoruba. The study collected data from 210 adults relating to 215 children at baseline and from 210 adults relating to 213 children at endline. These were different sampled populations.

Primary qualitative data were also collected at baseline and endline using topic guides. Sixteen FGDs (8 per ward) were carried out in each period. Respondents were community members (young women/men and older women/men), and usually 6–8 in a group. Adults who were caregivers or involved in the immunization decision-making relating to a child were included in the FGDs. Research assistants recruited participants with the help of community mobilizers.

A total of 25 key informants consisting of frontline health workers, policy-makers, local government implementers, religious and traditional leaders, and WDC and SMC members were recruited for SSIs at baseline and endline using purposive sampling. Additionally, SSIs were conducted with the 24 JAC members at endline. These were carried out to determine whether the PAR approach worked in the context and with the planned implementation structures and processes. The SSIs and FGDs lasted about 60 and 90 minutes, respectively, and were audio-recorded with the respondents’ consent.

### Data analysis

**Fig. 2:**
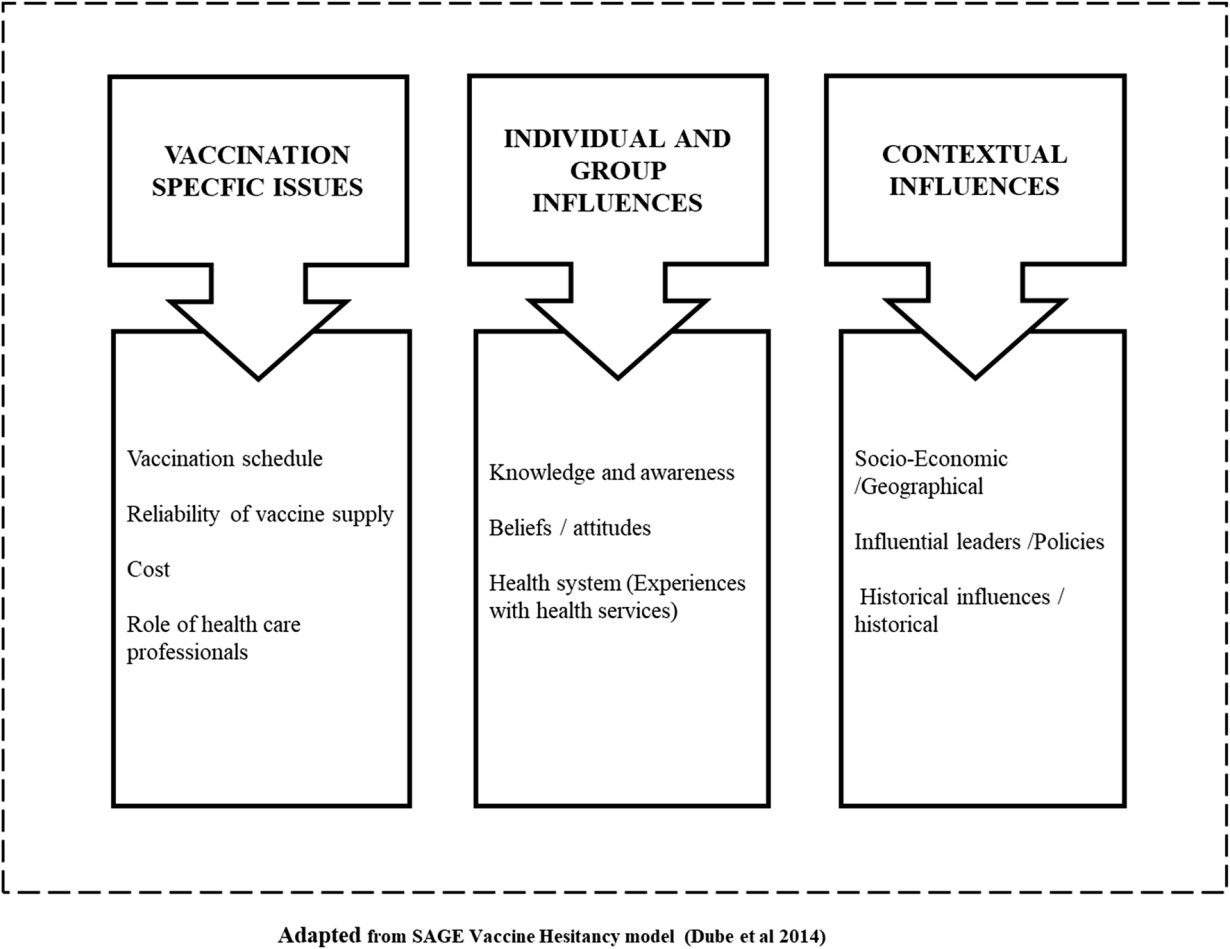
Conceptual framework

We used the theoretical framework for vaccine hesitancy by the WHO Strategic Advisory Group of Experts (SAGE) [23] to understand the PAR outcomes and explore the differences between baseline and endline. The model mapped the determinants of vaccine hesitancy in 13 countries and describes attitudes towards vaccination as a continuum ranging from complete acceptance to total refusal. It differentiates between contextual, individual, group and vaccine/vaccination-specific factors that influence immunization acceptance and utilization. We regrouped our study outcomes according to the themes in the hesitancy framework, and only the outcomes that emerged from the study were included in the adapted framework. For instance, we did not include the design of vaccine programme delivery (see Fig. 2). We explored whether immunization utilization had changed and the main drivers of change.

*Primary quantitative* data were analysed using SPSS [Statistical Package for the Social Sciences] version 21 software. The primary study outcome was immunization completeness. This was assessed as three doses of DPT/pentavalent vaccine as well as measles and yellow fever recorded as administered in an immunization card. To assess the association between covariate factors and immunization coverage, a univariate analysis was carried out for each factor and immunization coverage. All statistically significant factors/variables from the univariate analysis were included in a multivariate logistic regression model. Crude odds ratios were determined for each variable.

*Primary qualitative* data were analysed using NVivo 11. The primary outcomes assessed were changes in access to and utilization of immunization services. An inductive approach and open thematic coding were used. Transcripts were read by two qualitative researchers, coded and common themes and sub-themes identified according to the research objectives. A third qualitative researcher coded a few transcripts in order to ratify the codes and themes/sub-themes identified.

### Results

The respondent characteristics are described first. Then the outcomes of the PAR are presented according to the conceptual framework. Findings are compared and contrasted between different groups and between Ilara and Ipara wards where possible.

### Respondent characteristics

*HH survey* These results profile, at endline, 213 children and their caregivers (210) studied across 210 households in the study area. Half of the sampled children were older than 2 years of age and almost 51% were male. Similarly, at baseline (a different sampled population), 215 children and their 210 caregivers were studied across 210 households in the study area. Most of the caregivers were Yoruba (89% at baseline and 83% at endline)—this reflected the general population ratio between indigenous people and migrants. Table 2 details a comparison of gender, religious affiliation and socioeconomic characteristics including educational level of the respondents in both wards—in the baseline and endline surveys—and shows that the samples are comparable.

**Table 2:** Respondent characteristics—household survey

Variables		Caregivers' background characteristics (*N* = 420)							
		Baseline (*N* = 210)				Endline (*N* = 210)			
		Ilara (*n* = 86)		Ipara (*n* = 124)		Ilara (*n* = 83)		Ipara (*n* = 127)	
		Count	%	Count	%	Count	%	Count	%
Religion	Christianity	74_a_	86.0%	99_a_	79.8%	69_a_	83.1%	104_a_	81.9%
	Islam	7_a_	8.1%	22_b_	17.7%	10_a_	12.0%	22_a_	17.3%
	Others	5_a_	5.8%	3_a_	2.4%	4_a_	4.8%	1_a_	0.8%
Ethnicity	Yoruba	75_a_	87.2%	113_a_	91.1%	64_a_	77.1%	113_b_	89.0%
	Others	11_a_	12.8%	11_a_	8.9%	19_a_	22.9%	14_b_	11.0%
Employed	Yes	16_a_	18.6%	26_a_	21.0%	17_a_	20.5%	24_a_	18.9%
Highest level of education	None/preschool	22_a_	25.6%	11_b_	8.9%	6_a_	7.2%	7_a_	5.5%
	Primary	31_a_	36.0%	42_a_	33.9%	18_a_	21.7%	26_a_	20.5%
	Secondary	29_a_	33.7%	61_b_	49.2%	51_a_	61.4%	73_a_	57.5%
	Higher	4_a_	4.7%	10_a_	8.1%	8_a_	9.6%	21_a_	16.5%
Literacy	Cannot read at all	30_a_	34.9%	39_a_	31.5%	30_a_	36.1%	36_a_	28.3%
	Able to read only parts of sentence	21_a_	24.4%	23_a_	18.5%	15_a_	18.1%	27_a_	21.3%
	Able to read whole sentence	32_a_	37.2%	61_a_	49.2%	38_a_	45.8%	64_a_	50.4%
	Other	3_a_	3.5%	1_a_	0.8%	0^1^	0.0%	0	0.0%
Age of respondent (years)	< = 20	10_a_	11.6%	8_a_	6.5%	3_a_	3.6%	5_a_	3.9%
	21–30	34_a_	39.5%	50_a_	40.3%	37_a_	44.6%	55_a_	43.3%
	31–40	26_a_	30.2%	53_a_	42.7%	26_a_	31.3%	55_a_	43.3%
	41–50	10_a_	11.6%	10_a_	8.1%	13_a_	15.7%	10_a_	7.9%
	> = 51	6_a_	7.0%	3_a_	2.4%	4_a_	4.8%	2_a_	1.6%

Variable		Baseline (*N* = 215)				Endline (*N* = 213)			
		Ilara (*n* = 88)		Ipara (*n* = 127)		Ilara (*n* = 84)		Ipara (*n* = 129)	
Sex of child	Female	42_a_	47.7%	61_a_	48.0%	38_a_	45.2%	67_a_	51.9%
	Male	46_a_	52.3%	66_a_	52.0%	46_a_	54.8%	62_a_	48.1%
Age of child (months)	0–11	23_a_	26.1%	28_a_	22.0%	21_a_	25.0%	29_a_	22.5%
	12–23	23_a_	26.1%	36_a_	28.3%	21_a_	25.0%	34_a_	26.4%
	24–59	42_a_	47.7%	63_a_	49.6%	42_a_	50.0%	66_a_	51.2%

Values in the same row not sharing the same subscript are significantly different at *p* < 0.05

^1^Tests are adjusted for all pairwise comparisons (within a row) using the Bonferroni correction

*FGDs* The characteristics of the FGD participants at endline were similar to those at baseline. The participants were separated into different groups based on age and gender—the young men and women groups consisted of participants who were aged 18 to 39 years. The older women and men groups consisted of participants aged 40 to 65, with an outlier of 73. There were more Christians than Muslims. All the participants but one (a widow) were married.

*SSI* At endline, a total of 24 PAR participants (12 in each ward) were interviewed in Ilara and Ipara. Sixteen out of these 24 JAC members interviewed were female.

Interviews were also carried out with 25 key informants—policy-makers, local government officials, health workers and key community stakeholders involved in immunization service delivery. Six health workers were interviewed at endline compared with four at baseline, due to deployments that had taken place during the past year. Two WDC members and two religious leaders (an imam and pastor) were interviewed in each ward. The foremost traditional leaders in the wards (Kabiyesi in Ilara and Baale in Ipara) were interviewed as well. 

### Outcomes of the PAR intervention

The outcomes of the PAR are presented by addressing the main question first—did immunization utilization change? Then we examine how and why, using the conceptual framework. We present the quantitative findings first, then the qualitative. Where there are differences between Ilara and Ipara, we highlight these.

### Changes in immunization utilization

The primary study outcome was immunization completeness—assessed as three doses of DPT/pentavalent vaccine as well as measles and yellow fever recorded as administered on an immunization card. According to the routine vaccination schedule in Nigeria, the final antigens (measles and yellow fever vaccines) are administered at 9 months of age. The analysis of immunization completeness in the HHS encompassed all children between 9 and 59 months who should have plausibly achieved this outcome. Only 56 children (32.6%) over 9 months of age (*n* = 172) at baseline had immunization cards available for inspection. Availability of cards for assessment improved at endline (and was statistically significant) to 88 (52.4%) of 168 children over 9 months. At endline, assessment by card for children older than 9 months revealed a statistically significant increase from baseline (60.7%) (50% in Ilara, 67.7% in Ipara), to 90.9% (90.6% in Ilara, 91.1% in Ipara) (*p* < 0.05) of children having received all vaccinations (refer to Table 3 for details). However, when immunization status was assessed by card and recall, 146 (84.9%) of the 172 children over 9 months were assessed as fully immunized at baseline. At endline, similar figures—albeit a bit lower, but not statistically significant—of complete immunization were found, namely 136 (81.0%) of the 168 children older than 9 months.

**Table 3:** Immunization utilization in the wards, household survey

Variables		Immunization utilization children over 9 months of age (*N* = 340)											
		Baseline (*N* = 172)						Endline (*N* = 168)					
		Ilara (*n* = 69)		Ipara (*n* = 103)		Total		Ilara (*n* = 65)		Ipara (*n* = 103)		Total	
		Count	%	Count	%	Count	%	Count	%	Count	%	Count	%
Card availability	No cards	7_a_	10.1%	15_a_	14.6%	22_a_	12.8%	7_a_	10.8%	3_b_	2.9%	10_b_	6.0%
	Cards assessed	22_a_	31.9%	34_a_	33.0%	56_a_	32.6%	32_a_	49.2%	56_a_	54.4%	88_b_	52.4%
	Cards not assessed	40_a_	58.0%	54_a_	52.4%	94_a_	54.7%	26_a_	40.0%	44_a_	42.7%	70_b_	41.7%
Vaccination completed by card		(*n* = 22)		(*n* = 34)		(*n* = 56)		(*n* = 32)		(*n* = 56)		(*n* = 88)	
	No	11_a_	50.0%	11_a_	32.4%	22_a_	39.3%	3_a_	9.4%	5_a_	8.9%	8_b_	9.1%
	Yes	11_a_	50.0%	23_a_	67.6%	34_a_	60.7%	29_a_	90.6%	51_a_	91.1%	80_b_	90.9%
Vaccination completed by card and recall		(*n* = 69)		(*n* = 103)		(*n* = 172)		(*n* = 65)		(*n* = 103)		(*n* = 168)	
	No	15_a_	21.7%	11_b_	10.7%	26_a_	15.1%	18_a_	27.7%	14_b_	13.6%	32_a_	19.0%
	Yes	54_a_	78.3%	92_b_	89.3%	146_a_	84.9%	47_a_	72.3%	89_b_	86.4%	136_a_	81.0%

Values in the same row not sharing the same subscript are significantly different at *p* < 0.05

^1^Tests are adjusted for all pairwise comparisons within a row using the Bonferroni correction

The consensus in the FGDs was that immunization utilization by caregivers in Ipara and Ilara for their children had improved in the past year. Most policy-makers and local government officials commented that the coverage data from Remo North now showed fewer red and yellow indicators, indicating that the number of unimmunized children was declining and immunization-seeking behaviour had increased. The NHMIS categorizes access and utilization of immunization with numbers and colour codes ranging from 1 (deep green) for good access/utilization to 4 (red) for poor access/utilization.

Ilara has moved from category 4 to 2 now on routine immunization (RI). It’s very encouraging. —LGA official 2– PAR participant, Ilara

### Vaccination-specific factors

Caregivers in the HHS were asked about their most recent immunization visit (see Additional file 1). Significantly more caregivers visited fixed government health facilities for immunization services at endline (83.2%) than at baseline (54.2%) (*p* < 0.05). Also, a significantly higher proportion of caregivers in Ipara (88.7%) accessed routine immunization at fixed government facilities than in Ilara (75%) (*p* < 0.05) at endline. Interestingly, there was higher utilization of mobile or outreach services at baseline (34.8%) than at endline (10.6%). In terms of indirect costs, significantly more caregivers in the HHS were of the opinion that services were much cheaper (38.1%) at endline than at baseline (16.2%).

Intensified efforts on community mobilization with the JAC/WDC members were highlighted during the FGDs, and healthcare professionals were described as more motivated in carrying out community mobilization. This appeared to have encouraged greater facility use. According to the young women in both wards, health workers had become more responsive to the communities’ immunization needs during the past year—immunization was carried out on time, and greater availability of health workers and vaccines was noted. According to an LGA official:

…In the past, community members always complained of the attitude of the health workers—that they were too harsh and not accommodating. It’s not like that now. —LGA official 1– PAR participant, Ipara

Some of the respondents in the FGDs also attributed improvements in immunization utilization to improved relationships between the community members and health workers which had resulted from the dialogues and action.

Improved availability of vaccines for routine immunization was frequently mentioned in the discussion groups and by a majority of the SSI respondents in both wards; however, the perception of indirect costs of immunization remained the same at endline. Young women in Ilara and Ipara frequently reported that they still contributed 100 naira (approximately US$ 0.30) towards the transportation cost of the vaccines from the LGA headquarters to the wards.

### Individual and group influences

There was evidence of knowledge and awareness of immunization and its value. In the HHS, at baseline, similar to endline, the majority (95.7% and 96.1%) of caregivers stated that immunization prevents diseases, with polio and measles being the vaccine-preventable diseases that they were most aware of. Health facilities were the predominant sources of information on child health (91%), similar to baseline (91%). Provision of information on immunization was reported to be the most important function of the WDC by a little over 40% of respondents.

The survey findings were supported by the findings in the FGDs. A notable difference at endline was that the young women groups in both wards spoke more knowledgeably about immunization and contributed more to the discussions than at baseline. Young men and women groups in both wards also reported adverse events following immunization (AEFI) as an important reason why some people refused to take immunization for their children.

The SMC was adjudged to be the most active in immunization via mobilization of the communities. At endline, there were more frequent reports from the young women in both wards about passing on information about immunization to their neighbours. Leaders of the non-indigenous groups were reported to provide information on immunization to their groups in both wards. This information usually related to the dates and times of immunization, the value of immunization and information on AEFI. The content of the information was provided by the health workers, and the language barrier was overcome by the use of these mediators.

SSI respondents frequently reported improved attitudes of caregivers towards immunization, and this was also the general view in the FGDs. An important reason given for this improved attitude in both wards was reduced fear regarding AEFI. Participants reported that this was due to intensified health talks on AEFI given to mothers during facility visits and outreaches in the past year. JAC members in Ilara also stated that home visits by health workers, especially in the course of tracking defaulters, provided opportunities for the husbands to be educated on AEFI. Nevertheless, some young women in both wards still commented that AEFI was distressing and discouraging:

Respondent (R)1: Going for immunization doesn’t take anything. It’s just the issues that arise after. Like the sleepless nights. Not being able to sleep till morning (because of children crying from the pain at the immunization sites or fever).

R3: Truly, immunization is good for children. The only issue is that the arm injected gets swollen and is filled with pus. Why is that? —Young women, Ilara

Several key achievements relating to the overall health services in the past year were reported by PAR participants and FGD groups in Ilara. They included renovation of the health facility and reinstitution of antenatal care and delivery services. These were credited as the main reason for improved utilization of health and immunization services by caregivers. In Ipara, the provision of water supply in the health facility and delivery services for women in their first pregnancies were major achievements reported by the participants. Changes in the health services reported by SSI respondents and during the FGD are summarized in Table 4.

**Table 4:** Perceived changes in health services

Ilara	Ipara	Statements
At baseline, the majority of the community members in Ilara had expressed great dissatisfaction with their health facility—it had poor environmental conditions, functioned poorly and did not provide antenatal and delivery services. At endline, bushes around the facility had been cleared and chemicals sprayed to impede their growth; the toilet in the facility had been fixed; temporary measures to ensure water availability had been instituted. More health workers and midwives had been deployed to the health facility, and final-year medical students on their community medicine posting were also deployed to the facility; antenatal and delivery services had commenced and drugs were frequently reported as now available in the facility. Health workers were described as very responsive to the community members, especially the young mothers, and many expressed their utilization of the health and immunization services as a result of a “revitalized” health facility	At baseline, community members in Ipara were more (but not completely) satisfied with their health facility. Young mothers in Ipara were happy with delivery services but wanted the facility to be upgraded to also take deliveries for women in their first pregnancies. At endline, many stakeholders in Ipara reported that the health facility now takes deliveries for women in their first pregnancies	Formerly, we only have one staff available here, and after two weeks, we won’t see the staff again and the facility will be locked, but now thing aren’t like that anymore. Whenever you come it is either you meet one or two or three persons on duty —Young woman, Ilara One woman in my house fell sick around 1.00 am; they took her to the health centre and they attended to her. If it was before, as at last year, it was not like that; but we thank God for the relationship between the joint action committee and the community, it brings about good results. —Older man, Ilara
Health workers were now usually found at the health centre, and in a rare event where they were not available, the JAC community members were notified and the issue addressed. The health workers were described as being so responsive to the community members that they sometimes followed up pregnant women through home visits	Many respondents in Ipara reported greater availability of health workers and larger numbers as well. However, some older men and women asked for more “professionals” to be sent to their centre	R: Health workers are not enough. Where there are supposed to be three people doing a job, we find only one person I: Do you always meet the ones available on ground? R: Yes, but they are not enough —Older women, Ipara
At endline, a recurring area of discontentment frequently mentioned by many respondents in the FGD and IDI in Ilara was the issue of a water source in the health facility. This was a target in the Ilara JAP that had not been met due to financial constraints. All the measures used at endline to obtain water in the health facility were temporary and were reported as usually funded by the chairman JAC and the health workers	At baseline, unavailability of running water in the Ipara health facility was a source of discontentment among many community respondents. By endline, an arrangement had been made to route water from the borehole in the adjacent compound (belonging to the Ipara Development Committee) to the health centre, so water was now available in the facility. This development, which was an achieved target in the Ipara JAP, was frequently mentioned by Ipara respondents in the FGD and IDI as a source of joy to community members and the health workers	The issue of water is already solved. We have been able to connect water to the health facility, and with that, health workers are happier and mothers are happier to know that they would not have to bring buckets of water to the facility —Chairman JAC, Ipara

### Contextual influences

Multivariate logistic regression was performed for children over the age of 9 months to identify factors associated with completion of immunization based on assessment of cards and recall. Statistically significant factors were location (ward) and caregiver education (see Table 5). The likelihood of complete immunization for children older than 9 months in Ipara was 2.72 (CI 1.45–5.11, *p* = 0.002) compared with children in Ilara. Caregivers with a higher level of education were 5.09 times (CI 1.32–19.62, *p* = 0.018) as likely to fully immunize their children as uneducated caregivers. This trend continued: caregivers with secondary and primary education were respectively four times (CI 1.66–9.64, *p* = 0.002) and 2.93 times (CI 1.19–7.24, *p* = 0.02) as likely as uneducated caregivers to fully immunize their children.

**Table 5:** Multivariate logistic regression results on determinants of complete immunization, household survey

Variable	Vaccination coverage by card and recall (children older than 9 months)							
	(*N* = 340)							
	Category	Count	% Complete immunization	OR	SE	95% CI (OR)		*p* value
						Lower	Upper	
Intervention								
	Baseline	146	84.9%					
	Endline	136	81.0%	0.564	0.337	0.291	1.093	0.090
Location								
	Ilara	101	75.4%					
	Ipara	181	87.9%	2.719	0.320	1.451	5.095	0.002*
Chid age group (months)								
	9–11	12	92.3%					
	12–23	91	79.8%	0.378	1.099	0.044	3.258	0.376
	24–59	179	84.0%	0.488	1.091	0.058	4.138	0.511
Sex of child								
	Female	139	82.2%					
	Male	143	83.6%	1.020	0.319	0.546	1.907	0.951
Caregiver's employment status								
	No	51	79.7%					
	Yes	229	84.2%	1.106	0.399	0.506	2.415	0.801
Caregiver's highest level of education								
	None/preschool	26	61.9%					
	Primary	79	83.2%	2.930	0.461	1.187	7.236	0.020*
	Secondary	147	87.0%	3.997	0.449	1.658	9.639	0.002*
	Higher	30	88.2%	5.093	0.688	1.322	19.624	0.018*
Wealth quintile								
	Poorest	55	83.3%					
	Poor	64	87.7%	1.558	0.523	0.559	4.345	0.397
	Average	54	79.4%	0.626	0.476	0.247	1.592	0.326
	Rich	54	85.7%	1.000	0.530	0.354	2.828	1.000
	Richest	52	82.5%	0.691	0.511	0.254	1.881	0.469

*p < 0.05 (indicates statistical significance at a 5% level of significance)

The SMC and WDC are historical influences in terms of their collaboration with the immunization sector. The JAC continued with this collaboration in the implementation of the JAPS. Caregiver knowledge regarding who the SMC members were increased significantly at endline, from 20.5% to 48.1%, and their knowledge of WDC members also increased significantly, from 27.1% to 52.4%. Similar figures were reported across wards. Multivariate logistic regression showed that children over 9 months of age were 3.68 times as likely to be fully immunized when caregivers had knowledge of SMC members (CI 1.44–9.46).

### Spillover effects

**Fig. 3:**
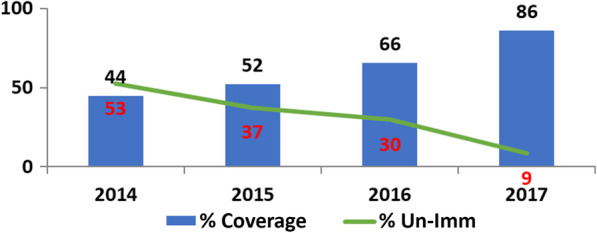
Trend of immunization uptake in Remo North

The involvement of the local government implementers resulted in a spillover of the strategies used in the JAPs into other wards in Remo North. Monitoring data showed that functional WDCs increased from 100 pre-PAR to 164 post-PAR. Correspondingly, immunization coverage in Remo North increased from 66% in 2016 to 86% in 2017, and the proportion of unimmunized children dropped from 30% to 9% (see Fig. 3). While the trend in immunization uptake cannot be attributed solely to the PAR, it appears that the intervention was an important contributor to the acceleration of the upward trend from 2016 to 2017 in Remo North.

## Discussion

Joint planning, implementation and evaluation of health interventions by community members, frontline health workers and local government officials improved the delivery and utilization of routine immunization. The increase in coverage assessed by cards only at endline for children over 9 months (90.9% compared with 60.7% at baseline) shows the effectiveness within this group. This is also encouraging because significantly more cards were available for inspection at endline—an important step in demonstrating utilization. In similar studies, Crowley et al. [10] found that the involvement of frontline workers in PAR helped a medical center improve influenza vaccination rates, and Ma et al. [24] highlighted significant increases in vaccination rates in the intervention group as a result of the contributions of the multiple partners in their community-based participatory research.

This study shows that collection of evidence and integration of evidence into discussion/dialogue with stakeholders can lead to change. Leveraging existing structures and potentials enhanced effectiveness: the PAR was decision-maker led, and embedded into the existing national immunization programme; the dialogues and action were also integrated into existing community social mobilization structures (WDC and SMC). All these appeared to have facilitated the acceptance, feasibility and implementation of the approach. These findings are in line with a review by Tetui et al. which noted that integrating PAR into systems reinforced local capacity and increased organizational support for the approach [25].

The HHS showed that a significantly greater number of caregivers visited fixed government health facilities for immunization services at endline. This might be explained by the joint actions resulting from the PAR in the area of health systems strengthening. This was especially clear regarding the revitalization of the Ilara health facility and reinstitution of antenatal care and delivery services. These were reported as the most important drivers of immunization utilization in the ward. This finding of health facility utilization as a driver of immunization use is consistent with that from other studies in Nigeria [26–29], Ethiopia [30, 31], Kenya [32] and other contexts [33, 34].

It is important to note the association between immunization utilization and the education of the mother in this study. Caregivers with a higher level of education were 5.44 times as likely to fully immunize their children as uneducated caregivers. This reflects the findings in similar studies in Nigeria [24, 35]. This association was stronger at endline than at baseline and displays a need to include more uneducated caregivers in subsequent interventions and incorporate more actions specifically targeting this group.

It is notable that within 8 months of implementation of the JAPs, important strides were made in immunization cards, health workers and vaccine availability during routine immunization in both wards. It seemed that the process of dialogue and reflection enabled the PAR participants to envision solutions to some long-standing problems, using resources which were already available. This quick turnaround and the spontaneous spillover of actions across Remo North supports the prospect that more health system barriers may be overcome if relevant stakeholders reflect together with a focus on finding solutions using existing resources. We are aware that external facilitation has its limitations in these types of processes, but research shows that it can motivate the participants to perform better. Tetui et al. [24] found in their review that the supportive monitoring from external researchers and partners helped to build local capacity and ensured quality.

### Limitations of the study

We used the NHMIS to track immunization coverage in this study but we were limited by issues related to completeness and accuracy of data. Triangulation with primary quantitative and qualitative data provided better insight. However, because we were keen on understanding perspectives on immunization among mothers of under-five children broadly, we did not limit the immunization completeness assessments in the survey to children aged 11–23 months, thereby reducing the precision of estimates of immunization coverage. We expect that the increased scope of understanding across the broader age group compensated for any loss in immunization completeness precision.

Poor availability of immunization cards (which featured more prominently at baseline than endline) was also a constraint to achieving an accurate assessment of immunization utilization in the surveys.

Furthermore, since children under 5 years of age were taken into consideration, there is a likelihood of recall bias—caregivers may not have recalled the number of immunization doses with precision, and figures given may have approximated immunization commencement rather than completion.

Recruitment of the respondents for the FGDs was done in collaboration with the community leaders/gatekeepers. As a result, some respondents may have given socially desirable answers in questions relating to immunization utilization.

## Conclusion

The PAR resulted in contextual solutions to problems identified by communities in both Ilara and Ipara. Collection of evidence and integration of evidence into discussions/dialogues with stakeholders can lead to change. Embedding the PAR into the National Programme on Immunization and integrating it into existing structures provide opportunities for a sustainable process of improving routine immunization, but the role of external facilitation has to be noted. There is a need for a longer period of implementation accompanied by research to gain a deeper understanding of the mechanisms by which the PAR worked.

## Supplementary Information

**Additional file 1.** Caregivers’ perceptions of most recent immunization visit, HH Survey.

## Data Availability

The data that support the findings of this study are available from the Ogun State Primary Health Care Development Board (SPHCDB) in Nigeria, the Royal Tropical Institute in the Netherlands and the grant funders. The data are available upon reasonable request and with the permission of the Ogun SPHCDB, and will be made publicly available in due course.

## Abbreviations

AEFI: Adverse events following immunization
DPT: Diphtheria-pertussis-tetanus
FGD: Focus group discussion
HHS: Household survey
LGA: Local government area
NHMIS: National Health Management Information System
REW: Reaching Every Ward
RI: Routine immunization
SMC: Social mobilization committee
SSI: Semi-structured interview
SAGE: WHO Strategic Advisory Group of Experts
WDC: Ward development committee
