# Supplementary material for: Using participatory action research to improve immunization utilization in areas with pockets of unimmunized children in Nigeria
Source: Health Res Policy Syst. 2021 Aug 11;19(Suppl 2):88. doi: 10.1186/s12961-021-00719-9 (PMC8356382; doi:10.1186/s12961-021-00719-9)
Supplement: Supplementary file 1 — Additional file 1. Caregivers’ perceptions of most recent immunization visit, HH Survey. [file 12961_2021_719_MOESM1_ESM.docx]

## **Additional File 1**

**Caregivers’ perceptions of most recent immunization visit, HH Survey**

| Children (0-24 months) | | | | | | | | | | | | | |
| --- | --- | --- | --- | --- | --- | --- | --- | --- | --- | --- | --- | --- | --- |
| Variable | | Baseline (N=108) | | | | | | Endline (N=103) | | | | | |
|  |  | Ilara (n=45) | | Ipara (n=63) | | Total | | Ilara (n=42) | | Ipara (n=61) | | Total | |
|  |  | Count | N % | Count | N % | Count | N % | Count | N % | Count | N % | Count | N % |
| Time since last immunization | <= 1 year | 40_a_ | 88.9% | 53_a_ | 84.1% | 93 | 86.1% | 34_a_ | 81.0% | 58_a_ | 95.0% | 92 | 89.1% |
|  | >1 year | 0^1^ | 0.0% | 0_a_ | 0.0% | 0 | 0.0% | 6_a_ | 14.3% | 3_a_ | 4.9% | 9 | 8.7% |
|  | No response | 5_a_ | 11.1% | 10_a_ | 15.9% | 15 | 13.9% | 2_a_ | 4.8% | 0_a_ | 0.0% | 2 | 1.9% |
| All Children (0-59 months) | | | | | | | | | | | | | |
| Variable | | Baseline (N=210) | | | | | | Endline (N=210) | | | | | |
|  |  | Ilara (n=86) | | Ipara (n=124) | | Total | | Ilara (n=83) | | Ipara (n=127) | | Total | |
|  |  | Count | N % | Count | N % | Count | N % | Count | N % | Count | N % | Count | N % |
| Place of last immuization visit | No Response | 9_a_ | 10.5% | 3_b_ | 2.4% | 12 | 5.7% | 2_a_ | 2.4% | 0^1^ | 0.0% | 2 | 1.0% |
|  | Fixed site Govt health facility | 46_a_ | 53.5% | 67_a_ | 54.0% | 113 | 53.8% | 63_a_ | 75.0% | 110_b_ | 88.7% | 173 | 83.2% |
|  | Fixed site Private health facility | 2_a_ | 2.3% | 2_a_ | 1.6% | 4 | 1.9% | 3_a_ | 3.6% | 8_a_ | 6.5% | 11 | 5.3% |
|  | Outreach/ mobile sites | 26_a_ | 30.2% | 47_a_ | 37.9% | 73 | 34.8% | 16_a_ | 19.0% | 6_b_ | 4.8% | 22 | 10.6% |
|  | Other | 3_a_ | 3.5% | 5_a_ | 4.0% | 8 | 3.8% | 0^1^ | 0.0% | 0^1^ | 0.0% | 0 | 0.0% |
| Opinion on distance to the immunization site | No Reponse | 5_a_ | 5.8% | 2_a_ | 1.6% | 7 | 3.3% | 4_a_ | 4.8% | 2_a_ | 1.6% | 6 | 2.9% |
|  | Very Short | 53_a_ | 61.6% | 51_b_ | 41.1% | 104 | 49.5% | 50_a_ | 59.5% | 49_b_ | 38.9% | 99 | 47.1% |
|  | Short | 15_a_ | 17.4% | 50_b_ | 40.3% | 65 | 31.0% | 16_a_ | 19.0% | 50_b_ | 39.7% | 66 | 31.4% |
|  | Average | 8_a_ | 9.3% | 11_a_ | 8.9% | 19 | 9.0% | 7_a_ | 8.3% | 13_a_ | 10.3% | 20 | 9.5% |
|  | Long | 2_a_ | 2.3% | 9_a_ | 7.3% | 11 | 5.2% | 5_a_ | 6.0% | 9_a_ | 7.1% | 14 | 6.7% |
|  | Very long | 3_a_ | 3.5% | 1_a_ | .8% | 4 | 1.9% | 2_a_ | 2.4% | 3_a_ | 2.4% | 5 | 2.4% |
| Opinion on direct cost of services | No Response | 6_a_ | 7.0% | 2_b_ | 1.6% | 8 | 3.8% | 3_a_ | 3.6% | 2_a_ | 1.6% | 5 | 2.4% |
|  | Free | 56_a_ | 65.1% | 73_a_ | 58.9% | 129 | 61.4% | 36_a_ | 42.9% | 34_b_ | 27.0% | 70 | 33.3% |
|  | Very Cheap | 13_a_ | 15.1% | 21_a_ | 16.9% | 34 | 16.2% | 23_a_ | 27.4% | 57_b_ | 45.2% | 80 | 38.1% |
|  | Cheap | 4_a_ | 4.7% | 20_b_ | 16.1% | 24 | 11.4% | 15_a_ | 17.9% | 20_a_ | 15.9% | 35 | 16.7% |
|  | Average | 4_a_ | 4.7% | 3_a_ | 2.4% | 7 | 3.3% | 3_a_ | 3.6% | 8_a_ | 6.3% | 11 | 5.2% |
|  | Expensive | 3_a_ | 3.5% | 5_a_ | 4.0% | 8 | 3.8% | 3_a_ | 3.6% | 5_a_ | 4.0% | 8 | 3.8% |
|  | Very Expensive | 0^1^ | 0.0% | 0^1^ | 0.0% | 0 | 0.0% | 1_a_ | 1.2% | 0^1^ | 0.0% | 1 | .5% |
| Rating of transportation costs | No Response | 4_a_ | 4.7% | 1_a_ | .8% | 5 | 2.4% | 3_a_ | 3.6% | 3_a_ | 2.4% | 6 | 2.9% |
|  | Free | 58_a_ | 67.4% | 73_a_ | 59.3% | 131 | 62.7% | 51_a_ | 60.7% | 66_a_ | 52.4% | 117 | 55.7% |
|  | Very Cheap | 7_a_ | 8.1% | 17_a_ | 13.8% | 24 | 11.5% | 20_a_ | 23.8% | 32_a_ | 25.4% | 52 | 24.8% |
|  | Cheap | 8_a_ | 9.3% | 22_a_ | 17.9% | 30 | 14.4% | 6_a_ | 7.1% | 11_a_ | 8.7% | 17 | 8.1% |
|  | Average | 3_a_ | 3.5% | 9_a_ | 7.3% | 12 | 5.7% | 2_a_ | 2.4% | 7_a_ | 5.6% | 9 | 4.3% |
|  | Expensive | 5_a_ | 5.8% | 1_b_ | .8% | 6 | 2.9% | 2_a_ | 2.4% | 7_a_ | 5.6% | 9 | 4.3% |
|  | Very Expensive | 1_a_ | 1.2% | 0^1^ | 0.0% | 1 | .5% | 0^1^ | 0.0% | 0^1^ | 0.0% | 0 | 0.0% |
| Opinion on behavior of health workers | No Response | 6_a_ | 7.0% | 1_b_ | .8% | 7 | 3.3% | 3_a_ | 3.6% | 2_a_ | 1.6% | 5 | 2.4% |
|  | Very helpful | 31_a_ | 36.0% | 50_a_ | 40.3% | 81 | 38.6% | 45_a_ | 53.6% | 52_a_ | 41.3% | 97 | 46.2% |
|  | Helpful | 42_a_ | 48.8% | 59_a_ | 47.6% | 101 | 48.1% | 32_a_ | 38.1% | 49_a_ | 38.9% | 81 | 38.6% |
|  | Neutral | 6_a_ | 7.0% | 8_a_ | 6.5% | 14 | 6.7% | 2_a_ | 2.4% | 14_b_ | 11.1% | 16 | 7.6% |
|  | Not helpful | 1_a_ | 1.2% | 3_a_ | 2.4% | 4 | 1.9% | 1_a_ | 1.2% | 8_a_ | 6.3% | 9 | 4.3% |
|  | Difficult | 0^1^ | 0.0% | 3_a_ | 2.4% | 3 | 1.4% | 1_a_ | 1.2% | 1_a_ | .8% | 2 | 1.0% |
| Availability of vaccines | No Response | 4_a_ | 4.7% | 1_a_ | .8% | 5 | 2.4% | 5_a_ | 6.0% | 2_a_ | 1.6% | 7 | 3.3% |
|  | Yes | 81_a_ | 94.2% | 117_a_ | 94.4% | 198 | 94.3% | 74_a_ | 88.1% | 117_a_ | 92.9% | 191 | 91.0% |
|  | No | 1_a_ | 1.2% | 6_a_ | 4.8% | 7 | 3.3% | 5_a_ | 6.0% | 7_a_ | 5.6% | 12 | 5.7% |
| Informed on what to expect at home after vaccination | No Response | 6_a_ | 7.0% | 1_b_ | .8% | 7 | 3.3% |  |  |  |  |  |  |
|  | Yes | 75_a_ | 87.2% | 118_b_ | 95.2% | 193 | 91.9% | 68_a_ | 84.0% | 121_b_ | 96.8% | 189 | 91.7% |
|  | No | 5_a_ | 5.8% | 5_a_ | 4.0% | 10 | 4.8% | 13_a_ | 16.0% | 4_b_ | 3.2% | 17 | 8.3% |
| Told what to do if the child had a problem at home | No Response | 5_a_ | 5.8% | 1_b_ | .8% | 6 | 2.9% |  |  |  |  |  |  |
|  | Yes | 76_a_ | 88.4% | 118_a_ | 95.2% | 194 | 92.4% | 69_a_ | 85.2% | 118_b_ | 94.4% | 187 | 90.8% |
|  | No | 5_a_ | 5.8% | 5_a_ | 4.0% | 10 | 4.8% | 12_a_ | 14.8% | 7_b_ | 5.6% | 19 | 9.2% |
| Adequately informed about where the child could be vaccinated | No Response | 5_a_ | 5.8% | 2_a_ | 1.6% | 7 | 3.3% |  |  |  |  |  |  |
|  | Yes | 75_a_ | 87.2% | 118_b_ | 95.2% | 193 | 91.9% | 74_a_ | 91.4% | 121_a_ | 96.8% | 195 | 94.7% |
|  | No | 3_a_ | 3.5% | 4_a_ | 3.2% | 7 | 3.3% | 6_a_ | 7.4% | 3_a_ | 2.4% | 9 | 4.4% |
|  | Don't know | 3_a_ | 3.5% | 0^1^ | 0.0% | 3 | 1.4% | 1_a_ | 1.2% | 1_a_ | .8% | 2 | 1.0% |
| Adequately informed about when the child needed to be vaccinated | No Response | 6_a_ | 7.0% | 1_b_ | .8% | 7 | 3.3% |  |  |  |  |  |  |
|  | Yes | 72_a_ | 83.7% | 119_b_ | 96.0% | 191 | 91.0% | 73_a_ | 90.1% | 122_b_ | 97.6% | 195 | 94.7% |
|  | No | 3_a_ | 3.5% | 2_a_ | 1.6% | 5 | 2.4% | 7_a_ | 8.6% | 2_b_ | 1.6% | 9 | 4.4% |
|  | Don't know | 5_a_ | 5.8% | 2_a_ | 1.6% | 7 | 3.3% | 1_a_ | 1.2% | 1_a_ | .8% | 2 | 1.0% |
| Note: Values in the same row and subtable not sharing the same subscript are significantly different at p< .05 in the two-sided test of equality for column proportions. Cells with no subscript are not included in the test. Tests assume equal variances.^2^ | | | | | | | | | | | | | |
| 1. This category is not used in comparisons because its column proportion is equal to zero or one. | | | | | | | | | | | | | |
